# Recycling 115,369 mobile phones for gorilla conservation over a six-year period (2009-2014) at Zoos Victoria: A case study of ‘points of influence’ and mobile phone donations

**DOI:** 10.1371/journal.pone.0206890

**Published:** 2018-12-05

**Authors:** Carla A. Litchfield, Rachel Lowry, Jill Dorrian

**Affiliations:** 1 Leader Conservation Psychology and Applied Animal Behaviour Research Group, School of Psychology, Social Work and Social Policy, University of South Australia, Magill, Australia; 2 Director Wildlife Conservation and Science, Zoos Victoria, Parkville, Australia; 3 Director Body, Brain, Behaviour Centre, University of South Australia, Magill, Australia; Deakin University - Melbourne Burwood Campus, AUSTRALIA

## Abstract

More than seven billion mobile phones are estimated to be in service globally, with more than a billion older phones likely to be retired. A major barrier to a sustainable circular economy for mobile phones is people’s hoarding of their retired phones. Old mobile phones may be refurbished for re-use or ultimately dismantled for possible extraction of elements, including ‘conflict’ metals such as coltan (containing elements tantalum and niobium), mined in eastern Democratic Republic of Congo and threatening wild populations of eastern Grauer’s gorillas (*Gorilla beringei graueri*). Zoos Victoria cares for western gorillas (*Gorilla gorilla gorilla*) who served as ambassadors for their Grauer’s gorilla counterparts in this community-based social marketing initiative. Through tracking of barcodes on satchels of recycled mobile phones, efficiency of ten different points of influence could be calculated for the ‘*They’re Calling on You*’ mobile phone recycling community campaign at Zoos Victoria in Australia. Over a six-year period (2009–2014), a total of 115,369 mobile phones were donated. The Courier Collect initiative resulted in 50,883 mobile phone donations (44% of total), followed by the Static Display at Melbourne Zoo, resulting in 29,778 mobile phone donations (26% of total). The number of phones collected for Keeper Talks (at Melbourne Zoo and Werribee Open Range Zoo) was 12,684 (11% of total), and in terms of fostering close connections between visitors and the conservation campaign, keeper talks were effective as one phone was donated for every four people attending a keeper talk at Werribee Open Range Zoo and one phone was donated for every 28 people who attended a keeper talk at Melbourne Zoo. We provide suggestions for future campaigns, so that accurate data capture can allow cost-benefit analyses to be conducted. Our results demonstrate that a conservation-based organisation, in partnership with corporate sponsors and community groups can effectively influenced people’s mobile phone recycling behavior, paving the way for international collaborations to maximize scale and impact.

## Introduction

One of the biggest challenges for conservation remains in how to translate people’s intention to act or pledges to act into actual behavior, and necessity for ongoing campaigns to ensure people continue engaging in the desired behavior. Indeed, many conservation problems are ‘wicked problems’, with climate change as a ‘super wicked problem’ [1]. As with many conservation programs, it is difficult to measure the success of a program and always somewhat subjective. For mobile phone recycling, the behavior of upgrading or replacing a mobile phone and the donation of the unwanted mobile phone by individuals occurs relatively infrequently, sometimes yearly [2], but globally such upgrades amount to an estimated 400 million unwanted mobile phones every year [3]. Linking mobile phone recycling behaviour directly to conservation outcomes for gorillas and other species impacted by mining for materials contained in mobile phones has not yet been attempted. Systematic analyses and evaluations of conservation program successes or failures in the field are uncommon, especially in regions where it is politically or logistically difficult to conduct scientific or community-based studies [4].

About 7.3 billion mobile phones were in service globally at the end of 2014, with sales reportedly [5]: 125 million for Apple (2009–2013), 364 million for Samsung (2006–2014), and 1 billion for Nokia (2003–2009). An estimated 1.32 billion mobile phones are in use in China [6]. Barriers to sustainable global supply chains of mobile phones (circular economy) include secrecy surrounding ingredients or material composition [7], and lack of recycling of more than one billion ‘retired’ mobile phones [8, 9]. E-waste recycling facilities do not exist in many countries [8], but when available (e.g. take-back schemes, recycling for refurbishment and re-use), most people ‘hoard’ or ‘hibernate’ their unwanted phones [10, 11]. Understanding why people do not recycle their mobile phones is essential, and we will return to this issue subsequently.

Annual sales of mobile phones and computers account for about 4% of gold and silver, and about 20% of cobalt and palladium mined globally [12]. Some materials used in mobile phones are known as ‘conflict metals’ as profits gleaned from mining them illegally support armed conflict and human rights abuses [13, 14], and include: coltan (containing elements tantalum & niobium), cassiterite (containing element tin), wolfram (containing element tungsten), and gold, sometimes known collectively as 3TG (Tantalum, Tungsten, Tin & Gold). Mining for coltan in eastern Democratic Republic of Congo (DRC) takes place in protected areas (e.g. Kahuzi-Biega National Park), outside regulatory frameworks, and threatens endangered species, such as eastern lowland gorillas, and their habitats [15, 16]. In 2009, an estimated 300 gorillas were killed for bushmeat in southern DRC, with hunting and illegal wildlife trade taking place in and around mining sites [16]. In eastern DRC, recent population estimates of Grauer’s gorilla (*Gorilla beringei graueri*) show a dramatic 77–93% decline, as they also do for the eastern chimpanzee (*Pan troglodytes schweinfurthii*) with a 22–45% decline [17]. As a result, the IUCN Red List of Threatened Species (IUCN) has classified Grauer’s gorillas as Critically Endangered, with an estimated 3,800 remaining in the wild [18]. However, small populations of gorillas at the periphery of their range in DRC are isolated in forest fragments, with as few as six gorillas remaining in Mt, Tshiaberimu, and just over 200 gorillas left in the high-altitude sector of Kahuzi-Biega National Park [19].

The United Nations, governments and mobile phone manufacturers are working to establish certified ‘DRC conflict free’ trading chains [7, 20, 21]. After recycling, if mobile phones are unable to be refurbished or reused, elements including ‘conflict metals’ can be recovered, thereby potentially decreasing mining in gorilla habitats in DRC. Gold and silver content in a single mobile phone may be as high as US$1.83, but recovery, processing and refining of metals to achieve high levels of purity (>99%) is costly and may be harmful to the environment [22]. Nevertheless, the high value of gold, platinum, silver and palladium is the prime economic incentive for materials recovery from mobile phones [22]. For every 30–40 mobile phones that are recycled, on average 1 gram of gold can be recovered (e.g. from the printed circuit board), which equates to a whole ton of alluvial ore being mined and processed from natural sources [23]. Just as mobile phone sales are soaring, and gold content is increasing in some smartphones (e.g. iPhone 6 contains 30mg of gold), natural sources of gold are expected to ‘run out’ by 2030 [24].

**Fig 1:**
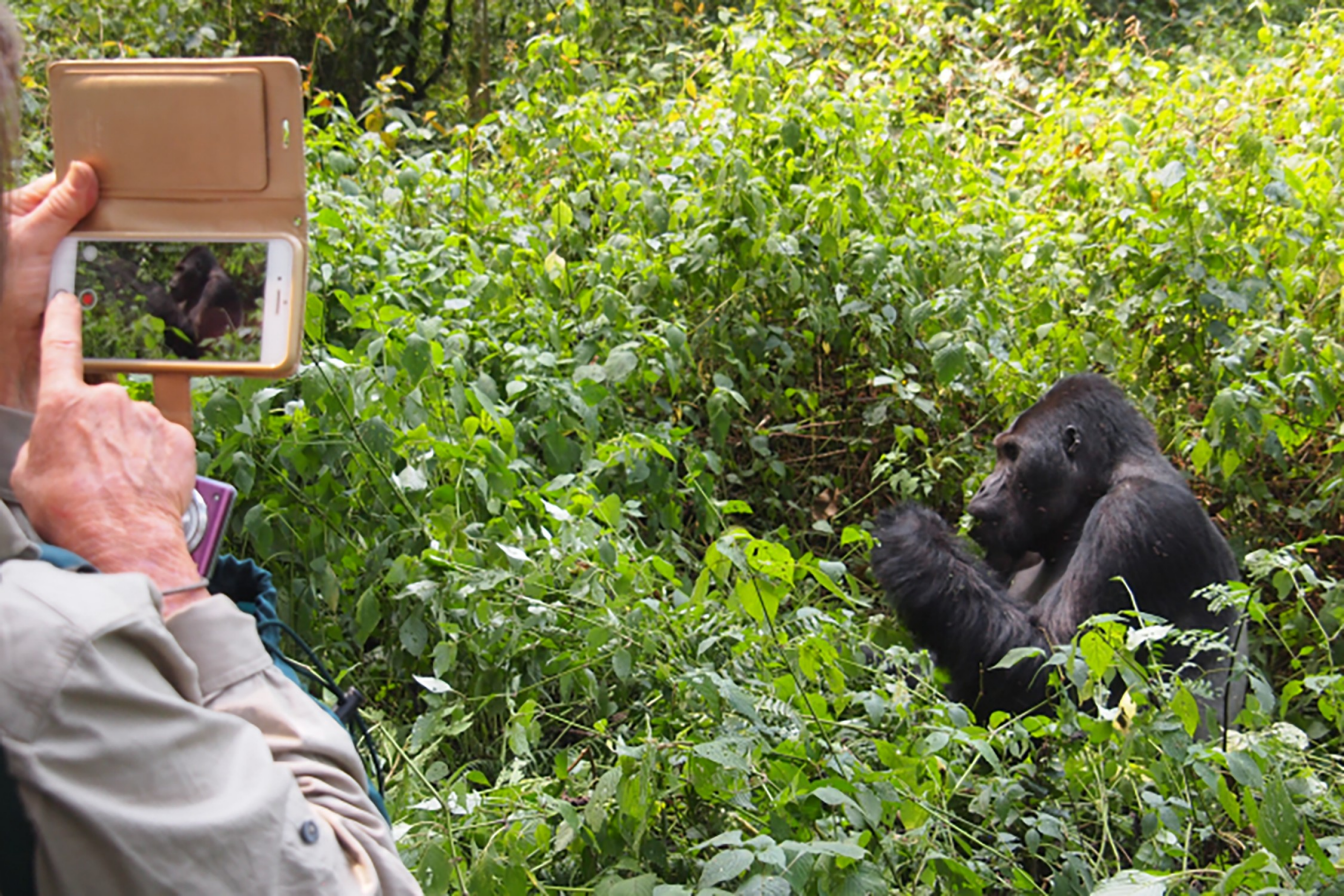
Australian tourist photographing Chimanuka on a smartphone as part of a gorilla tourism experience at Kahuzi-Biega National Park in DRC in July 2017.

For extraction of elements from old mobile phones to occur, people must recycle their unwanted phones. That is, behavior of individual mobile phone consumers is essentially the key to successful mobile phone donation campaigns or programs. Tourists use their mobile phones to record gorilla encounters in DRC, near illegal mines, sometimes traveling around the world to catch a glimpse of silverback Chimanuka and his family (Fig 1).

### Hoarding or storage of unwanted mobile phones at home

Globally, most people do not recycle their unwanted mobile phones, instead disposing of them incorrectly with their household waste, or most often, ‘hibernating’ [25] or ‘hoarding’ these mobile phones in a drawer or cupboard at home [26]. This hoarding behaviour is problematic, as it hinders recycling efforts, and occurs across regions, irrespective of the wealth of a country and its citizens (e.g. United Kingdom, [11]; Kenya, [27]; Sri Lanka, [28]; Australia, [29]; Philippines, [30]; Norway, [31]; Japan, [10]; Switzerland & Liechtenstein [25]; Spain [32]; Austria [33]; Nepal [34]). Mobile phones are also hoarded by people in China [3], with higher rates reported for smartphones [35]. Despite an increase in variety of mobile phone recycling options available in China, including reverse vending machines in subway stations and shopping malls, where people can return a mobile phone in exchange for an agreed upon discount coupon for future purchase [6], almost 80% of people store their unwanted mobile phones at home [35].

Results from surveys and interviews of mobile phone consumers have reported reasons for hoarding mobile phones as: future use or spare parts, data storage, toys for children, nostalgia [25], emotional attachment [32], and for smartphones, concerns over data security (theft of personal/private information) after recycling [35]. Recently, the concept of a secondary phone has emerged, with some people keeping a retired but still functioning phone as a ‘sacrificial device’ for occasions when their new phone might become lost or damaged (e.g. holidays, nights out), or as a ‘time capsule’ of memories or ‘life narratives’ (e.g. data stored as photos, texts) [36].

Psychological motivations for replacing (e.g. perceived technological or aesthetic obsolescence, social pressure, desire for novelty [33]) and hoarding (e.g. emotional attachment [32]; fear of access to personal data [6]) mobile phones may differ and studies to date tend to include questions that focus on either one or the other, without use of standardised psychological scales or measures to allow exploration of psychological differences between individuals. Hoarding as a broader psychological concept of accumulating objects, including mobile phones, and becoming emotionally attached to them when they are essentially worthless may in extreme cases indicate psycho-pathology (e.g. Obsessive Compulsive Disorder) [36], while attachment anxiety has been reported in cases where people anthropormophize their smartphone, believing it to have emotions and consciousness [37]. People with a stronger tendency to hoard objects may not replace their mobile phones as often [36]. Hoarding is problematic since precious (including conflict) metals in unrecycled old mobile phones are not extracted and potentially returned to the supply loop, thereby reducing the need for mining in wilderness areas. In Germany, by 2035 it is predicted that more than 8,000 tonnes of precious (including conflict) metals will lie ‘in unrecycled mobile and smartphones [38], and in China, by 2025 an estimated nine tonnes of gold, 15 tonnes of silver and 3,107 tonnes of copper will also be out of the supply loop in 0.35 billion unrecycled mobile and smartphones [6]. Many people are likely to be unaware of the environmental/health risks associated with hoarding mobile phones, or the effect on conservation of habitats, species and even mineral resources [35]. Incorrect disposal of mobile phones is hazardous to humans, other animals and environmental health as mobile phones (comprised of components such as plastic, battery, liquid crystal display, printed circuit board) sent to landfill leach toxic metals or metalloids, and if burnt produce toxic dioxins and furans [39]. Even when awareness of environmental problems associated with waste/unrecycled mobile phones is high, this does not seem to translate into recycling behaviour [6].

### Zoos as facilitators of pro-conservation behaviors

Zoos Victoria is comprised of three properties: Melbourne Zoo, Healesville Sanctuary and Werribee Open Range Zoo, with a combined total of over 2.3 million annual visitors [40]. Parks and zoos as provide opportunities for interacting with nature, with effects on human well-being, social interactions and relationships with nature [41]. Conservation campaigns delivered within a zoo setting, may facilitate social change, since more than 600 million people globally are estimated to visit zoos every year [42]. There is a growing body of evidence that zoos can influence human behavior change [43, 44]. Since there is strong evidence to link the rise of electronic telecommunication devices to the dramatic decline of the Eastern Lowland Gorilla species within the Democratic Republic of Congo [15, 18], a global effort to recycle and refurbish electronic devices such as mobile phones is required [45].

Changing human behavior is challenging, since no single technique or behavior change tool works in all situations [46]. Information-intensive approaches or awareness-raising campaigns are often ineffective on their own in changing unsustainable human behavior [47]. In Australia, Zoos Victoria runs environmental education programs at its three properties (inside zoo) as well as community conservation campaigns (outside zoo and online) to tackle human-induced threats to wildlife [43]. In 2005, post-visit teacher calls were placed to almost 50 schools whose students had taken part in environmental education programs at Werribee Open Range Zoo, and it was discovered that not a single school had installed a nest-box or planted native grasses, and only one school had installed a frog bog incorrectly. As a result, to facilitate uptake of pro-conservation behaviors, Zoos Victoria developed the Connect-Understand-Act (CUA) conservation education model [48], provided as Supporting Information (S3 Appendix), which is based on principles of psychology (e.g. contemporary learning theories) and utilizes behavior change tools from Community-Based Social Marketing [49]. The CUA model [48] guided the design and development of the *They’re Calling On You* (TCOY) campaign to facilitate donation of retired mobile phones (target behavior) by mobile phone users over 12 years of age (target audience).

Gorillas were chosen as the species for this community-based social marketing initiative since Zoos Victoria cares for Western gorillas (*Gorilla gorilla gorilla*) who serve as ambassadors for their Grauer’s gorilla counterparts, impacted significantly by coltan mining. By focusing on gorillas, and the link between mining in DRC, and the importance of mobile phone recycling, the campaign was able to highlight both the topic of gorilla conservation and welfare.

**Fig 2:**
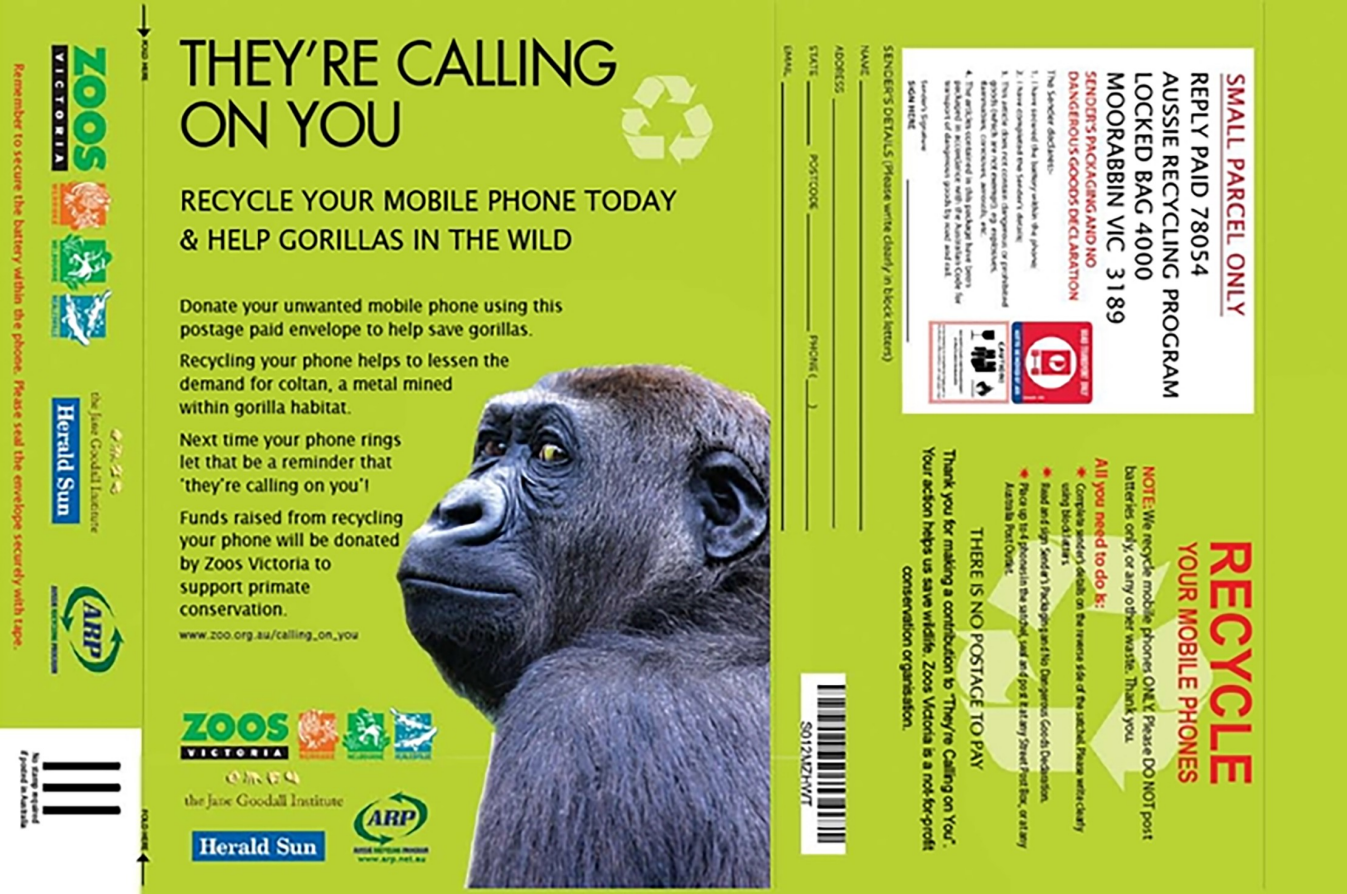
Artwork for TCOY satchel for return of mobile phones with image of Yakini on the front (left) and tracking barcode (for Herald Sun newspaper insert) and other information on the back (right).

A barriers versus benefits analysis [50] was conducted by Rachel Lowry prior to implementation through a workshop with the education, visitor experience and Melbourne Zoo primate keeper team. Since cost and inconvenience of ‘postage and packaging’ was identified as a potential barrier to people donating phones, pre-paid 50% recycled plastic satchels with a printed image of resident Melbourne Zoo gorilla, Yakini, were produced (Fig 2). These visually appealing bright green satchels acted as ‘prompts’ or reminders to recycle their phone, and satchels were recycled along with returned mobile phones. Partnering with Aussie Recycling Program (ARP) allowed funds from donated phones to be directed towards primate conservation, serving as an additional incentive for people to recycle their phones.

### Aims of case study and research questions

Our main aim was to evaluate the first six-years (2009–2014) of the ongoing ‘*They’re Calling on You’* (TCOY) community mobile phone recycling campaign developed and run by Zoos Victoria. Separate barcodes printed on recycling satchels allowed us to track different methods of connecting with the target audience (Points of Influence or POI), with number of mobile phones returned providing a basis for assessing efficiency of these POI.

In this paper we address the following research questions:

Q1: What was the total number of mobile phones donated to each POI?

Q2: Research Question 2: What was the potential participant exposure to each POI, costs associated with each POI, and estimated recoverable elements (including conflict elements) and landfill space saved?

A further aim was to use our findings to provide recommendations for accurate data capture, so that organisations can conduct cost-benefit analyses before they undertake or implement a similar campaign, similar to a Return on Investment [49]. For example, provision of pre-paid mobile phone recycling satchels has budgetary implications and may raise potential environmental concerns (e.g. waste of plastic). At the very least, organisations need to anticipate how many satchels (or other packaging) to produce and distribute. Widely disseminating these findings is important, as our data provide a way of measuring actual behavior (number of phones donated) rather than self-reported behavioral intention (questionnaire item), with people’s intention to act often not translated into action [51].

## Materials and methods

We collected data for ten Points of Influence (POI), inside the zoo and outside the zoo (wider community), made possible because each POI was allocated a separate barcode, printed on the recycling satchels, which were returned to Aussie Recycling Program (ARP) at Moorabbin in Victoria, Australia. ARP kept records for each barcode, and numbers of phones collected by courier. Monthly updates of mobile phone donations, according to separate barcodes, were provided by ARP to the General manager Community Conservation (Rachel Lowry) from October 2008 until December 2014, who maintained a central database at Zoos Victoria.

In 2008, Zoos Victoria trialled the mobile phone recycling program over three months (October- December), with 2,376 mobile phones successfully returned to Melbourne Zoo (329 through Keeper Talks at MZ, 502 through Static Display at MZ, 1,375 by Courier Collect & 171 by website reply label; see procedure for details of each POI). The total number of visitors to MZ during the trial period was 256,622, The Australasian Regional Association of Zoological Parks and Aquaria (renamed Zoo and Aquarium Association), adopted the program as a regional campaign throughout the Year of the Gorilla (2009) along with 13 other zoological institutions. Zoos Victoria considered this a successful pilot phase and committed to running the campaign on an ongoing basis.

**Fig 3:**
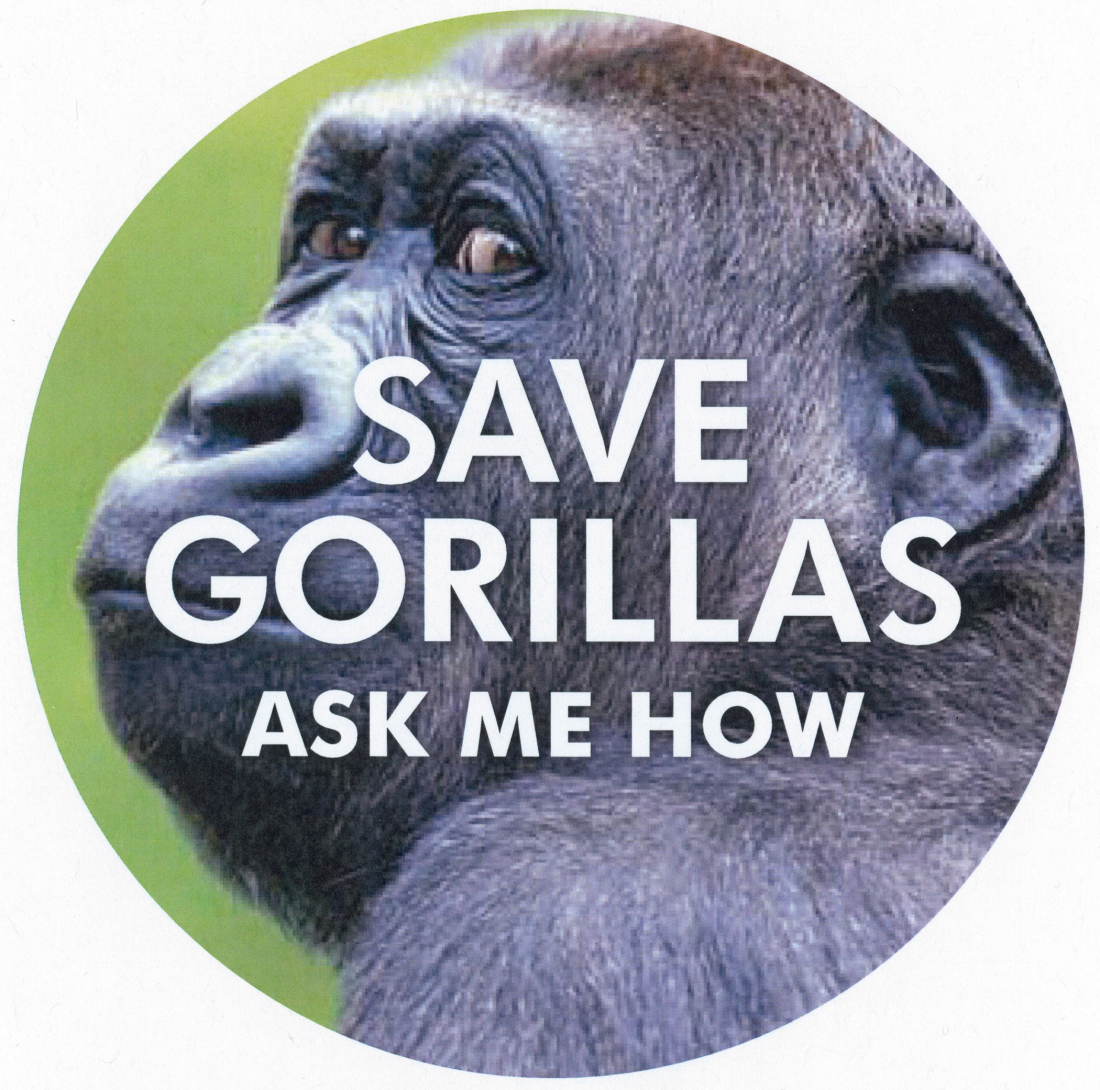
Artwork for staff uniform button badges work during the first year of the campaign.

Initially, four POI were developed, with others added as capacity became available, and further images are provided as Supporting Information (S4 Appendix). Throughout the first year of the campaign, all staff working at Melbourne Zoo wore badges as part of their uniform (Fig 3). The monthly number of visitors to Melbourne Zoo and Werribee Open Range Zoo were recorded at the entrances as part of Zoos Victoria normal operating procedure and are provided as Supporting Information (S1 Appendix).

### Inside zoo points of influence

#### Keeper talks at MZ and WORZ

**Participants:** Potential participants consisted of visitors who attended daily scheduled talk about gorillas, given by zoo keepers in the public viewing area outside the gorilla enclosure at MZ from January 2009 and at WORZ from August 2011. Although number of visitors at these talks was not recorded systematically, Zoos Victoria estimate that on average there were three daily talks attended by 35 people per talk (105 people per day) at MZ and one daily talk attended by 15 people at WORZ.

**Procedure:** Keeper(s) giving the talk about gorillas included information about the campaign and offered satchels (MZ satchel barcode S012MZFF; WORZ barcode S012WORZFF) to any person interested in recycling a mobile phone. Data are available for six years (January 2009- end December 2014) for MZ and three years and five months for WORZ (August 2011- end December 2014).

**Associated costs:** The cost of recyclable plastic satchels was covered by the Aussie Recycling Program, with batches of 20,000 printed at a time, at a cost of AUD $2000 (AUD 10 cents per satchel). Since keeper talks are a daily occurrence, no additional costs were incurred by Zoos Victoria.

#### Static display at MZ

**Participants:** Potential participants consisted of any visitor to MZ from January 2009, who walked past and noticed one of the static satchel displays.

**Procedure:** Zoo visitors could collect satchels from three dispensers, which were accompanied by signage about the campaign, located at the front and back exits at MZ (barcode S012MZPU). Data are available for six years (January 2009- end December 2014).

**Associated costs:** The cost of satchels was again covered by the Aussie Recycling Program, with batches of 20,000 printed at a time, at a cost of AUD $2000 (AUD 10 cents per satchel). The total cost to Zoos Victoria of three satchel dispensers was AUD $216. The dispensers were restocked daily, and minimal staff time was required to check and reorder another batch whenever satchel numbers in storage fell below 6000.

#### Other activities at MZ and WORZ

**Participants:** Potential participants consisted of any visitor attending special zoo events at WORZ from December 2012 and at MZ from March 2013.

**Procedure:** Zoo staff and volunteers present at special zoo events offered satchels to any person interested in recycling a mobile phone (WORZ barcodes S012WZOFF, S012WZON, & S012WZRR; MZ barcodes S012MZEVT, S012MZMEM, S012MZRR, & S012MZYATZ). Data are available for one year and ten months (March 2013- end December 2014) for MZ and two years and one month (December 2012- end December 2014) for WORZ.

**Associated costs:** The cost of satchels was again covered by the Aussie Recycling Program, with batches of 20,000 printed at a time, at a cost of AUD $2000 (AUD 10 cents per satchel). These events were run as part of Zoos Victoria’s business as usual operations and therefore no additional costs were incurred by Zoos Victoria.

### Outside zoo points of influence

#### Courier Collect

**Participants:** Potential participants consisted of any school, organisation or community group in the state of Victoria, Australia, which registered its interest with Zoos Victoria after hearing about the campaign from January 2009.

**Procedure:** As part of its promotion of the TCOY campaign, Zoos Victoria staff targeted local schools and community/corporate groups through keeper talks, email ‘news blasts’ to Zoos Victoria members, brochures, internal business newsletters, media articles, school discovery and learning programs and the Zoos Victoria website. When any participating group collected more than 10 mobile phones, they could ring either MZ (barcode MELZ001) from January 2009 or WORZ (barcode S012WORZPU) from September 2011 to arrange for a courier to collect the mobile phones. Satchels were not used. Instead, people were instructed to pack mobile phones into a box, print out a shipping label from the campaign website, fix it to the box, and a courier collected them later that day. Occasionally, school groups returned donated phones to MZ administration office, who then organised courier transfer to ARP. The number of courier collections was not recorded systematically, just the total number of mobile phones returned. By the end of 2013, the list of registered groups was 276 schools and 466 organisations, which provides a total figure of 742 community groups for the six-year period (2009–2014). Data are available for six years (January 2009- end December 2014) for MZ and two years and four months (September 2011- end December 2014) for WORZ.

**Associated costs:** For promotion of the campaign, Zoos Victoria staff contributed time and effort as part of their typical workload, particularly keeping staff, education staff, and members of the Corporate Sponsorship and Philanthropy team and Wildlife Conservation and Science team. The Aussie Recycling Program covered the costs of the courier collections (no figure for this cost is available).

#### Website reply paid labels

**Participants:** Potential participants consisted of any visitor to the Zoos Victoria TCOY campaign webpage from January 2009.

**Procedure:** After visiting the campaign webpage, participants could print off a label (barcode S012MZRPL), stick it onto an envelope/parcel containing the mobile phone to be returned, and post it to the Aussie Recycling Program at no cost to the participant. Zoos Victoria does not have access to data about monthly website visits for this period.

**Associated costs:** The cost of postage was covered by the Aussie Recycling Program (no figure for this cost is available), and the person donating the mobile phone bore the cost of printing the label and purchasing the envelope/parcel themselves.

#### Bendigo bank branches

**Participants:** Potential participants consisted of any client visiting one of the 235 Bendigo Bank branches in Victoria, Australia, from January 2009.

**Procedure:** Bendigo Bank offered its assistance after hearing about the TCOY program during discussions with the Zoos Victoria Corporate Sponsorship and Philanthropy team. Participants returned their mobile phones to Bendigo Bank branches (barcode S012MZBB), who then rang a courier to collect them, when more than 10 phones were returned. A few branches requested satchels (<1000 satchels; costs covered by Aussie Recycling Program) which were handed to any customer who requested one after seeing the sign promoting the campaign at the bank.

**Associated costs:** The cost of satchels was again covered by the Aussie Recycling Program (AUD 10 cents per satchel), as was cost of courier collection. For promotion of the campaign, Zoos Victoria and Bendigo Bank staff contributed time and effort as part of their typical workload.

#### *Herald Sun* newspaper

**Participants:** Potential participants consisted of any person who obtained a copy of the *Herald Sun* newspaper published on the 11th June 2011.

**Procedure:** The *Herald Sun* is the official print media partner of Zoos Victoria and promoted the TCOY campaign as part of its partnership commitment after being approached by the Zoos Victoria Corporate Sponsorship team. On the 11th June 2011, 539,000 satchels (barcode S012MZHWT) were distributed as inserts in the *Herald Sun* delivered across Victoria, Australia.

**Associated costs:** The cost of satchels was covered by the Aussie Recycling Program, at a cost of about AUD $53,900 (AUD 10 cents per satchel). For promotion of the campaign, Zoos Victoria and *Herald Sun* staff contributed time and effort as part of their typical workload.

## Results

### Research Question 1: What was the total number of mobile phones donated to each POI?

**Fig 4:**
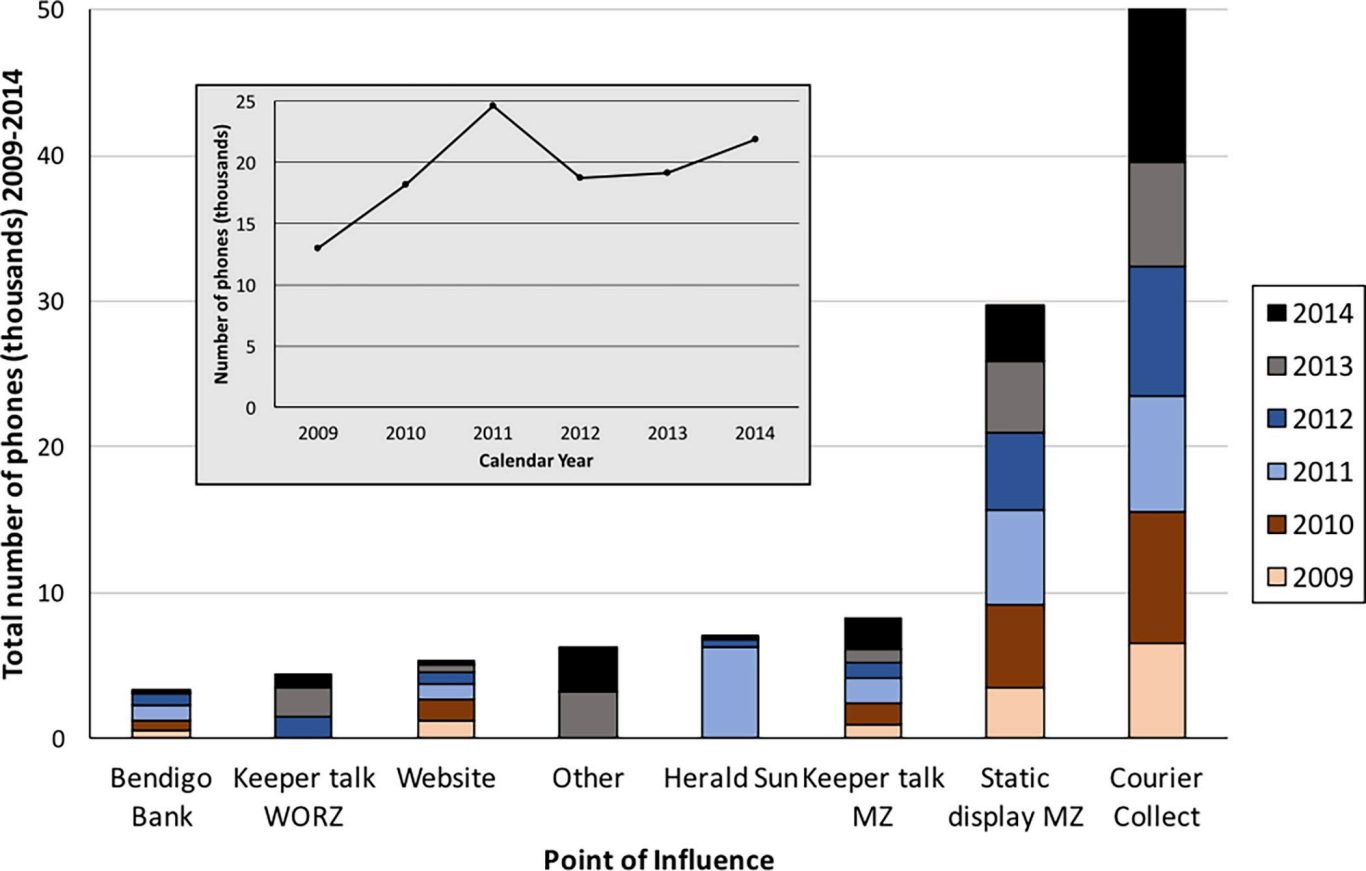
The total number of phones donated to each point-of-influence for the six-year period (2009–2014). The stacked column chart presents bar height as total number of phones for each POI and colored sections within each bar indicate the relative proportion of phones collected in each calendar year. POI is arranged from least (left) to most (right) phones collected. It should be noted that Melbourne Zoo Keeper Talks MZ, Static Display MZ, the Website, and Bendigo Bank POI were conducted throughout (from January 2014), the other POIs were conducted for only part of the 6-year period (as described in the procedure section). The grey inset shows total phones collected (y-axis) for each calendar year (x-axis).

The total number of phones donated from 2009–2014, as a number distributed to each point-of-influence, is provided in Fig 4 and the information as a table is provided as Supporting Information (S2 Appendix).

The total number of phones donated was 115,369. The largest proportion of these phones came from the Courier Collect initiative (50,883), accounting for 44% of the total, followed by the Static Display MZ (26%), with the remaining POI accounting for between 3% and 7% each (Fig 4). If the number of phones collected for Keeper Talks at MZ and WORZ are combined, then this POI accounts for more than 12,684 mobile phones (11% of total) being donated. The inset in Fig 4 shows that the number of phones collected peaked in 2011 with 24,574 phones, coincident with the addition of the *Herald Sun* POI.

### Research Question 2: What was the potential participant exposure to each POI, costs associated with each POI, and estimated recoverable elements (including conflict elements) and landfill space saved?

There were 7,003,488 visitors to MZ during the six-year period (January 2009- end December 2014) and 1,667,834 to WORZ during the same period, with monthly figures provided as Supporting Information (S1 Appendix). Table 1 summarizes, for each POI, the potential participant pool (number of people likely to have had exposure to each POI), satchel costs, other additional costs (arising over and above those incurred during normal operations), and the number of phones returned, with estimated recoverable elements and landfill space saved. Not all details are available for all POI. In particular, the Website POI is not included in the table due to lack of information. Of note, while the satchels were associated with cost, they were also associated with waste. In particular, in the *Herald Sun* campaign, the 539,000 satchels distributed yielded only a 1.3% return rate, wasting AUD $53,195 worth of satchels. In total, 3663kg of elements were recovered (182kg of conflict elements) [22], and returned phones were conservatively estimated to account for between 12 and 20 cubic metres. Further information related to our calculations of element recovery and landfill space saved are provided in Supporting Information (S5 Appendix).

**Table 1:** For each point of influence (POI), potential participants, costs, phones returned, and estimated recoverable elements and landfill space.

Point of Influence	Potential Participant Pool	Satchel Cost (10c/satchel)	Waste Satchel Cost	Other Additional Cost	Phones Returned	Recoverable Elements^	Landfill Space#
**Keeper Talk MZ**	talk attendees *n* = 230,055 6,573 talks 3 talks/day for 6y @35 people/talk	$3,000 ~30,000 satchels ~5,000/year*	$2,400 ~20% return rate*	Nil part of existing keeper talks	8,209	~261kg conflict~13kg	~0.82m^3^
**Keeper Talk WORZ**	talk attendees *n* = 18,705 1,247 talks 1 talk/day for 3y 5mo @15 people/talk	*Not available*	*Not available*	Nil part of existing keeper talks	4,475	~142kg conflict~7kg	~0.45m^3^
**Static Display MZ**	total zoo visitors for 6y *n* = 7,003,488	$33,000 ~330,000 satchels ~55,000/year*	$30,888 ~6.4% return rate*	$216 3 satchel dispensers	29,778	~945kg conflict~47kg	~2.98m^3^
**Courier Collect**	members of community groups = 742 schools = 276 organisations = 466	*Not used*	*Not used*	*courier service* *figure not* *available*	50,883	~1,616kg conflict~80kg	~5.09m^3^
**Bendigo Bank**	visitors to branches = 235	$100 <1,000 satchels	*Not available*	*courier service* *figure not* *available*	3,340	~106kg conflict~5kg	~0.33m^3^
**Herald Sun**	readers received satchels *n* = 539,000	$53,900 539,000 satchels	$53,195 531,953 not returned 1.3% return rate	*staff time for* *promotion figure* *not available*	7,052	~224kg conflict~11kg	~0.71m^3^

*complete yearly figures were unavailable–the total number of satchels and return rate are estimated from complete dataset from 2009

^^^estimated from Table X, with total conflict elements (Tantalum, Gold, Tin, Tungsten) at an average of 1.58g per phone, and other elements (Iron, Copper, Nickel, Zinc, Silver) at an average of 30.2 grams per phone

^#^conservatively estimated at 100cm^3^ per phone.

**Fig 5:**
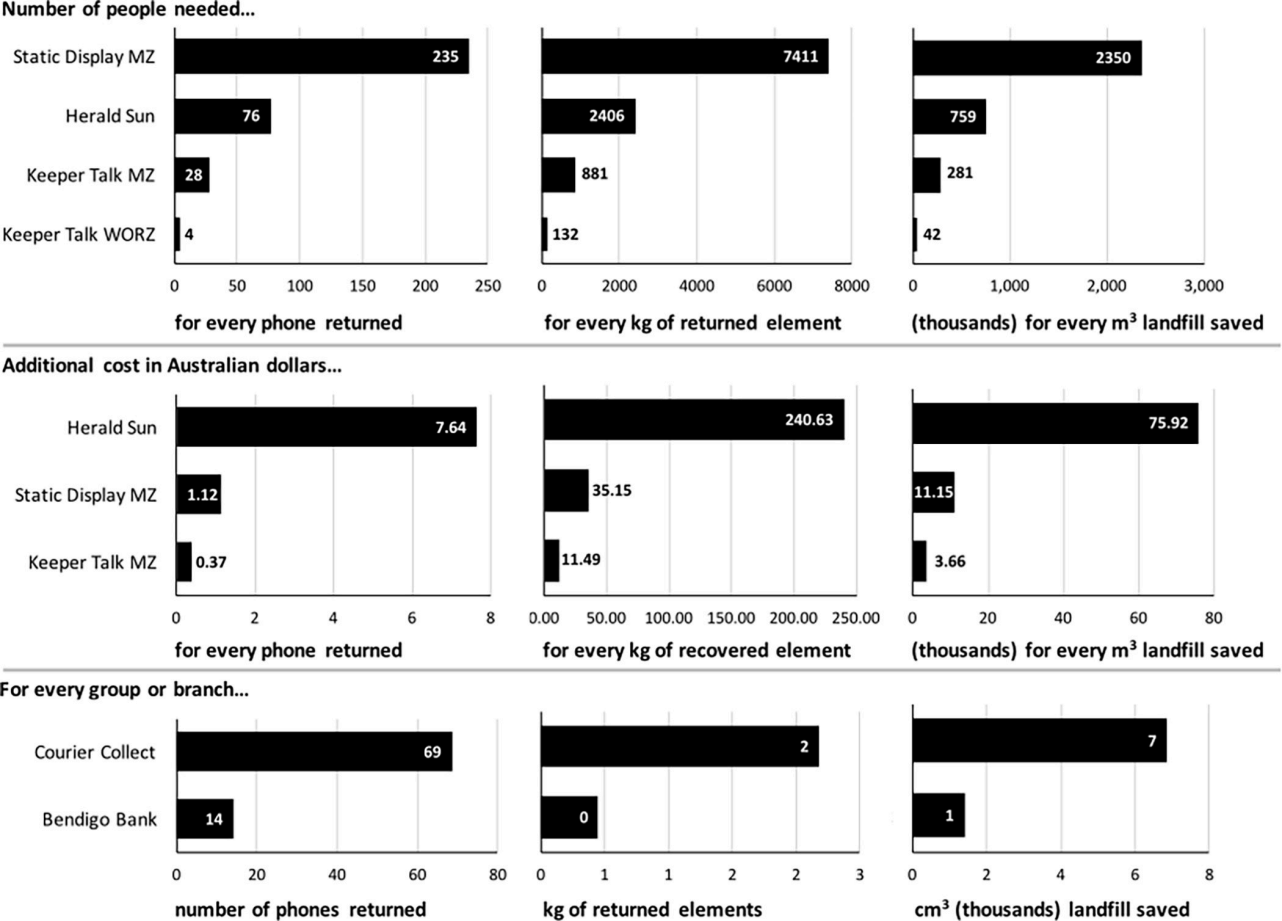
Example methods for comparing Points of Influence. ***Upper Panel*:** Comparison by estimated number of people required to collect each phone [people(*n*)/phones(*n*)], each kilogram (kg) of returned elements [people(*n*)/elements(kg)], and every cubic meter (m^3^) of landfill saved [people(*n*)/landfill(m^3^)]. ***Middle Panel*:** Comparison by estimated additional cost (i.e. above normal operations) in Australian dollars (AUD) required to collect each phone [AUD/phones(*n*)], each kilogram (kg) of recovered elements [AUD/elements(kg)], and every cubic meter (m^3^) of landfill saved [AUD(*n*)/landfill(m^3^)]. ***Lower Panel*:** Comparison of number of phones [phones(*n*)/groups(*n*)], kg of recovered elements [elements(kg)/groups(*n*)], and m^3^ of landfill saved [landfill(m^3^)/groups(*n*)] for every community group or bank branch. Estimations provided are dependent on available data and are calculated from information provided in Table 1 and Supporting Information (S5 Appendix).

Fig 5 provides an illustration of several techniques that could be used to compare the relative efficiencies of each POI for those with sufficient data (Table 1). Relative to the Static Display, with an estimated 235 people exposed for one phone returned, the *Herald Sun* required an estimated 76 people, and the Keeper Talks an estimated 28 (MZ) or 4 (WORZ) people per phone returned. These relative efficiencies can be expressed in terms of estimated kilograms of recovered elements, and cubic metres of landfill saved (Fig 5, Upper Panel). Relative to the Herald Sun POI, with an estimated extra cost of AUD $7.64 per phone, the Static Display was associated with an estimated $1.12, and the Keeper Talk (MZ) with an estimated AUD 37 cents per phone (Fig 5, Middle Panel). The Courier Collect POI yielded 69 phones for every group/organisation and the Bendigo Bank POI yielded 14 phones per branch (Fig 5, Lower Panel).

Therefore, while the Courier Collect and Static Display POI yielded the greatest number of phones, the Keeper Talks are relatively strong in terms of efficiency when considering the number of phones returned relative to the number of potential participants exposed, and the smaller additional cost for every phone returned.

## Discussion

In terms of efficiencies, Keeper talks are a cost-effective way of encouraging mobile phone donations, since they are given every day as part of the standard operating procedure. They are particularly effective for smaller groups of visitors (15 people) typically attending a keeper talk at WORZ, since one phone was donated for every four people. This means that more than a third of people attending a WORZ Keeper talk are inspired to act, suggesting that there is a connection to the conservation message and keeper as the source of information. Not surprisingly, at a busier urban zoo like MZ, where keeper talks are attended by twice as many people, and there are three per day, there is less potential to interact as closely with a keeper or perhaps even to hear the message clearly. The use of couriers to collect bulk numbers of mobile phones was clearly effective, did not require printing or potential waste of satchels, and potentially fostered close ties with the community outside the zoos, particularly with school groups. The static displays also required little effort in terms of staff support, and although not interactive in any way, they were efficient in influencing mobile phone donations.

### Recommendations for future data capture to allow accurate cost-benefit analyses

Cost/benefit comparison calculations in this paper were limited by the data collected for each POI. In order to facilitate a more complete comparison, Table 2 summarizes examples of information that would be required. This is not an exhaustive list but is provided to spur consideration for future research. Overall, time should be taken to design and implement data capture methods for the total number of potential participants who are exposed to each point of influence, any embedded and additional costs, and details of returns. Of note, research in this area would benefit from standardized techniques for quantifying the value of returned materials, especially element weights, and the associated monetary and environmental costs (or recovery).

**Table 2:** For each example point of influence suggestions are given for the information that is needed to evaluate and compare their cost/benefit (non-exhaustive list).

Point of Influence Examples	Number of Potential Participants	Embedded Costs	Additional Costs	Returns
**Zoo Keeper Talks**	Number of: • talks/time • visitors/talk	• Keeper time (training & delivery)	• Return mechanism (e.g. satchel) price & environmental impact • Participant time	• Number of phones • Point of influence Type of phone to estimate: • Size & contribution to landfill • recoverable elements (value in $ & in conflict reduction–we need clarity & consistency in published values to use in these calculations) These can all be expressed relative to: • potential participants • embedded &/or additional costs • wasted resources (e.g. satchels that were not returned)
**In Zoo Static** **Displays**	Number of: • zoo visitor numbers/time • displays • people passing each display/time	• Zoo staff time (training, placement, monitoring, & restocking displays)	• Display price & environmental impact • Return mechanism (e.g. satchel) price & environmental impact • Participant time	
**Courier Collect** **Programs**	Number of: • organisations • sites • members/organisation • Visitors/organisation site/time	• Organisation staff time • (training, placement of advertising materials, e.g., posters)	• Poster/flyer printing • Number of courier trips • Length of trips • Courier driver salary/trip • Driving costs/trip (e.g. fuel, wear & tear on vehicle, emissions)	
**Websites**	Number of: • Visits/time • Downloads of relevant materials	• Zoo staff time (training, questions regarding website campaign)	• Development of website content (salaries) • Construction & maintenance of website (salaries, domain costs, etc.) • Costs (money & time) to participants for access, downloading, & printing for return mechanism	
**Newspaper,** **Magazine** **Campaigns**	Number: • in circulation • sold/time	• Newspaper staff time for inclusion in print round	• Return mechanism (e.g. satchel) price & environmental impact • Participant time	

Moreover, embedding mechanisms for feedback from participants, as well as staff at zoos (and other participating organizations) would allow for continuous improvement to maximize the efficacy of any POI. This could involve quantitative surveys, but would ideally involve collection of qualitative data, to allow for novel and potentially unexpected suggestions for improvement. Taken together, this case study highlights the critical gains that could be afforded by zoo and community group partnerships with researchers, where evaluation methods can be discussed and co-designed prior to POI implementation.

## Conclusions

The results of this case study show that it is possible to move beyond awareness-raising campaigns about mobile phone recycling, or surveys reporting intention to donate unwanted phones, to collection of donated mobile phones through a conservation-based organisation. Although it may never be possible to show a direct effect of mobile phone donations on gorilla conservation in Africa, there have been improvements in gorilla and habitat protection at Kahuzi-Biega National Park, in the sector visited by tourists (highland Tshivanga Sector) [18]. Future improvements may indirectly be attributed to the pressure of international governments and organisations to regulate supply chains, such as the source of Coltan in the Democratic Republic of Congo [52], although due diligence processes in supply chain management are hampered by illegal conflict minerals becoming mixed-in with legally exported and mined minerals early on in the supply chain, and once refined into metals at smelters, it is almost impossible to determine an accurate source of origin [53].

Based on the outcomes of this campaign, the international zoo community can help facilitate mobile phone recycling behavior for the benefit of gorilla conservation if a global campaign is strategically activated across zoos (e.g. through the World Association of Zoos and Aquariums, WAZA). Recycling mobile phones and the process of extracting minerals from end-of-life mobile phones remains complex [45, 54], as scientists explore more environmentally-friendly (since some hydrometallurgical processes use toxic reagents like cyanide or thiosulphate) and efficient chemical and biological methods for mineral extraction [55]. Can the first six-years of the ongoing TCOY program be considered a success? The 117,745 mobile phones donated (including three-month trial period donations) may seem a ‘drop in the ocean’, representing 0.01% of the one billion phones that may be retired globally. However, for the State of Victoria in Australia, with an estimated population of under six million people [56], this figure is more impressive, and shows that a conservation-based organisation can effectively influence people to recycle their phones for gorilla conservation, paving the way for international collaborations to maximize scale and impact.

## Supporting information

S1 Appendix Data set for number of mobile phones donated monthly to Zoos Victoria from 2009–2014 and monthly zoo visitor numbers. (PDF)

S2 Appendix Data set for total number of phones donated yearly from 2009–2014. (PDF)

S3 Appendix The connect–understand–act model. (PDF)

S4 Appendix Images showing additional details of three points of influence. (PDF)

S5 Appendix Estimated amount of recoverable elements including conflict elements and landfill space saved. (PDF)
