# Supplementary material for: Recycling 115,369 mobile phones for gorilla conservation over a six-year period (2009-2014) at Zoos Victoria: A case study of ‘points of influence’ and mobile phone donations
Source: PLoS One. 2018 Dec 5;13(12):e0206890. doi: 10.1371/journal.pone.0206890 (PMC6281204; doi:10.1371/journal.pone.0206890)
Supplement: S1 Appendix — (PDF) [file pone.0206890.s001.pdf]

S1 Appendix

Number of mobile phones donated to Zoos Victoria on a monthly basis for every year from 2009-2014 for different points of influence for the *They're Calling on You* campaign and monthly zoo visitor numbers.

Table 1. Number of mobile phones donated to Zoos Victoria every month during 2009 for points of influence and monthly visitor numbers for Melbourne Zoo (MZ).

Point of influence	Jan	Feb	Mar	Apr	May	Jun	Jul	Aug	Sept	Oct	Nov	Dec	Total
**Inside Zoo**													
Keeper talk MZ	72	85	73	102	189	71	21	67	66	117	130	-	993
Static display MZ	367	179	163	299	395	384	398	234	470	362	268	-	3,519
**Outside Zoo**													
Courier Collect (MZ)	307	58	138	143	1,267	292	421	611	1,127	1,165	687	307	6,523
Website reply paid label	281	139	75	167	35	62	104	86	149	90	101	-	1,289
Bendigo bank	-	-	-	-	-	-	-	145	290	137	52	-	624
**Total donated**	1,027	461	449	711	1,886	809	944	1,143	2,102	1,871	1,238	307	12,948
**Visitors to MZ**	120,079	60,039	82,424	95,147	72,837	55,388	71,395	61,582	77,216	94,717	75,326	126,107	992,257

In 2009, highest number of mobile phones were donated through 'Static Display MZ' in January, February, March, April and June, whereas 'Courier Collect (MZ)' resulted in highest number of mobile phone donations for the other seven months, with more than 1,100 mobile phones donated through this point of influence in May, September and October (Table 1).

Table 2. Number of mobile phones donated to Zoos Victoria every month during 2010 for points of influence and monthly visitor numbers for Melbourne Zoo (MZ).

**Point of influence**	**Jan**	**Feb**	**Mar**	**Apr**	**May**	**Jun**	**Jul**	**Aug**	**Sept**	**Oct**	**Nov**	**Dec**	**Total**
**Inside Zoo**													
Keeper talk MZ	269	122	205	138	82	108	106	66	104	100	81	100	1,481
Static display MZ	854	498	707	494	311	358	401	390	350	455	433	393	5,644
**Outside Zoo**													
Courier Collect (MZ)	1,863	421	508	766	972	355	568	257	1,203	792	822	456	8,983
Website reply paid label	231	137	239	122	57	117	87	136	91	81	50	93	1,441
Bendigo bank	157	65	131	30	18	27	76	57	24	20	16	15	636
**Total donated**	3,374	1,243	1,790	1,550	1,440	965	1,238	906	1,772	1,448	1,402	1,057	18,185
**Visitors to MZ**	153,878	91,690	117,509	151,022	99,573	74,031	94,494	65,581	101,491	113,240	84,171	108,291	1,254,971

In 2010, highest number of mobile phones were donated through 'Static Display MZ' in February, March, June and August, and 'Courier Collect (MZ)' had highest number of mobile phone donations for the other eight months, with more than 1,800 mobile phones donated through this point of influence in January and about 1,200 in September (Table 2).

In 2011, highest number of mobile phones were again donated through 'Static Display MZ' in January, April and October (Table 3), with more than 1,000 mobile phones donated in March. 'Courier Collect (MZ)' had highest number of mobile phone donations for February, May and December, with more than 1,100 mobile phones donated in September and more than 1,600 in November. The *Herald Sun* campaign resulted in 3,775 mobile phones donations in the first month, almost 1,500 donated a month later, and this figure dropping to about 540 in the third month after this one-off newspaper campaign was launched.

Table 3. Number of mobile phones donated to Zoos Victoria every month during 2011 for points of influence and monthly visitor numbers for Melbourne Zoo (MZ) and Werribee Open Range Zoo (WORZ).

Point of influence	Jan	Feb	Mar	Apr	May	Jun	Jul	Aug	Sept	Oct	Nov	Dec	Total
**Inside Zoo**													
Keeper talk MZ	108	78	388	136	162	92	187	127	123	56	148	51	1,656
Keeper talk WORZ	-	-	-	-	-	-	-	6	10	23	12	5	56
Static display MZ	638	395	1,037	492	673	422	544	628	512	448	432	242	6,463
**Outside Zoo**													
Courier Collect (MZ)	504	568	391	212	685	584	501	777	1,177	176	1,614	638	7,827
Courier Collect (WORZ)	-	-	-	-	-	-	-	-	1	18	79	47	145
Website reply paid label	119	62	121	62	78	91	141	141	86	49	78	41	1,069
Herald Sun	-	-	-	-	-	3,775	1,473	541	234	107	153	47	6,330
Bendigo bank	27	28	19	3	20	4	120	311	193	139	127	37	1,028
**Total donated**	1,396	1,131	1,956	905	1,618	4,968	2,966	2,531	2,336	1,016	2,643	1,108	24,574
**Visitors to MZ**	146,332	60,180	80,718	116,624	56,141	67,182	109,333	83,695	89,004	109,815	79,252	105,401	1,103,677
**Visitors to WORZ**	44,034	16,424	24,909	35,301	15,222	17,941	36,098	20,819	26,729	34,491	29,382	42,132	343,482

In 2012, highest mobile phones donations were for ‘Static Display MZ’ in January, February, April, July and November, whereas ‘Courier Collect (MZ)’ resulted in highest mobile phone donations for the other seven months (Table 4), with more than 1,600 mobile phones donated through this point of influence in June and about 1,050 in October.

Table 4. Number of mobile phones donated to Zoos Victoria every month during 2012 for nine different points of influence and monthly visitor numbers for Melbourne Zoo (MZ) and Werribee Open Range Zoo (WORZ).

Point of influence	Jan	Feb	Mar	Apr	May	Jun	Jul	Aug	Sept	Oct	Nov	Dec	Total
**Inside Zoo**													
Keeper talk MZ	132	111	75	65	58	131	59	74	134	63	54	77	1,033
Keeper talk WORZ	63	128	115	161	119	102	122	128	133	137	127	100	1,435
Static display MZ	778	674	447	389	347	321	385	406	246	371	474	458	5,296
Other (WORZ)	-	-	-	-	-	-	-	-	-	-	-	6	6
**Outside Zoo**													
Courier Collect (MZ)	754	593	524	363	380	1,603	326	532	815	1,056	459	724	8,129
Courier Collect (WORZ)	180	89	84	99	37	35	116	38	46	21	18	22	785
Website reply paid label	107	61	42	59	60	50	96	54	65	56	59	63	772
Herald Sun	119	51	45	43	41	65	42	16	29	8	12	16	487
Bendigo bank	86	89	120	69	75	35	63	43	61	53	35	16	745
**Total donated**	2,219	1,796	1,452	1,248	1,117	2,342	1,209	1,291	1,529	1,765	1,238	1,482	18,688
**Visitors to MZ**	143,856	63,446	77,723	128,121	58,841	61,324	94,175	65,871	106,587	123,224	94,236	107,434	1,124,838
**Visitors to WORZ**	62,145	27,369	30,878	51,085	18,577	20,037	36,352	18,528	34,175	38,596	28,149	38,235	404,126

In 2013, about 1,100 mobile phones were donated at a Melbourne Zoo function ('Other MZ') in March (Table 5). 'Static Display MZ' resulted in highest mobile phone donations in February, June, September and October, and 'Courier Collect (MZ)' resulted in highest donations of mobile phones for the other seven months, including more than 1,000 mobile phones donated in April and November.

Table 5. Number of mobile phones donated to Zoos Victoria every month during 2013 for different points of influence and monthly visitor numbers for Melbourne Zoo (MZ) and Werribee Open Range Zoo (WORZ).

Point of influence	Jan	Feb	Mar	Apr	May	Jun	Jul	Aug	Sept	Oct	Nov	Dec	Total
**Inside Zoo**													
Keeper talk MZ	197	86	78	58	76	53	104	34	173	55	91	38	1,043
Keeper talk WORZ	174	169	84	156	253	161	211	67	259	244	87	84	1,949
Static display MZ	626	522	431	468	595	332	468	183	569	478	203	58	4,933
Other (MZ)	6	18	1,106	461	280	209	195	93	215	215	108	47	2,953
Other (WORZ)	-	-	30	76	36	12	17	6	27	11	7	-	222
**Outside Zoo**													
Courier Collect (MZ)	807	486	182	1,050	935	292	635	672	300	153	1,018	274	6,804
Courier Collect (WORZ)	-	-	-	-	-	99	6	10	26	68	57	35	301
Website reply paid label	76	52	55	38	19	58	69	46	92	20	30	6	561
Herald Sun	27	15	18	16	15	12	3	2	28	7	11	1	155
Bendigo bank	24	9	5	21	29	18	7	10	37	15	24	5	204
**Total donated**	1,937	1,357	1,989	2,344	2,238	1,246	1,715	1,123	1,726	1,266	1,636	548	19,125
**Visitors to MZ**	174,917	71,106	94,284	154,357	75,855	80,168	115,987	78,591	113,091	104,820	93,378	115,620	1,272,174
**Visitors to WORZ**	57,986	23,901	28,690	51,885	21,781	28,210	51,461	23,546	41,156	34,640	31,243	38,285	432,784

In 2014, 'Courier Collect (MZ)' resulted in highest monthly mobile phone donations, with almost 2,100 phones donated in January and about 1,500 in February (Table 6). However, in May more phones were donated for 'Static Display MZ'.

Table 6. Number of mobile phones donated to Zoos Victoria every month during 2014 for different points of influence and monthly visitor numbers for Melbourne Zoo (MZ) and Werribee Open Range Zoo (WORZ).

Point of influence	Jan	Feb	Mar	Apr	May	Jun	Jul	Aug	Sept	Oct	Nov*	Dec*	Total
**Inside Zoo**													
Keeper talk MZ	143	160	135	396	191	148	213	157	325	101	-	-	1,969
Keeper talk WORZ	94	53	78	235	87	115	103	45	99	62	-	-	971
Static display MZ	380	253	249	730	314	388	517	280	476	175	-	-	3,762
Other (MZ)	311	408	30	757	227	240	20	132	214	47	-	-	2,842
Other (WORZ)	6	8	3	14	-	3	3	74	-	-	-	-	120
**Outside Zoo**													
Courier Collect (MZ)	2,098	1,503	646	1,011	182	981	1,399	956	844	431	-	-	10,051
Courier Collect (WORZ)	133	138	60	228	163	96	156	-	91	38	-	-	1,103
Website reply paid label	54	52	10	23	0	2	63	8	11	12	-	-	235
Herald Sun	8	15	9	16	5	3	-	8	2	9	-	-	75
Bendigo bank	7	10	3	32	11	9	8	11	5	-	-	-	96
**Total donated**	3,234	2,600	1,507	3,442	1,180	1,985	2,663	1,671	2,067	875	-	*625	21,224
**Visitors to MZ**	150,041	61,165	98,382	125,966	84,116	70,672	98,271	103,023	125,067	119,798	100,422	118,648	1,255,571
**Visitors to WORZ**	65,226	25,276	36,512	56,466	28,716	24,800	36,890	33,047	51,164	47,445	36,783	45,117	487,442

* No points of influence were tracked in November and December (just total mobile phone donations) as the Recycling Partner was changed during this period
