# Supplementary material for: Recycling 115,369 mobile phones for gorilla conservation over a six-year period (2009-2014) at Zoos Victoria: A case study of ‘points of influence’ and mobile phone donations
Source: PLoS One. 2018 Dec 5;13(12):e0206890. doi: 10.1371/journal.pone.0206890 (PMC6281204; doi:10.1371/journal.pone.0206890)
Supplement: S2 Appendix — (PDF) [file pone.0206890.s002.pdf]

S2 Appendix

Total number of phones donated per year for 2009-2014 for different points of influence for the *They're Calling on You* campaign

<u>Table 1</u>. Number of mobile phones donated to Zoos Victoria from 2009-2014 for different points of influence.

Point of influence	2009	2010	2011	2012	2013	2014*	Total
**<u>Inside Zoo</u>**							
Keeper talk MZ	993	1,481	1,656	1,033	1,043	2,003	8,209
Keeper talk WORZ	-	-	56	1,435	1,949	1,035	4,475
Static display MZ	3,519	5,644	6,463	5,296	4,933	3,923	29,778
Other (MZ & WORZ)	-	-	-	6	3,175	3,066	6,247
**<u>Outside Zoo</u>**							
Courier (MZ & WORZ)	6,523	8,983	7,972	8,914	7,105	11,386	50,883
Website reply paid label	1,289	1,441	1,069	772	561	253	5,385
Herald Sun	-	-	6,330	487	155	80	7,052
Bendigo Bank	624	636	1,028	745	204	103	3,340
**Total donated**	12,948	18,185	24,574	18,688	19,125	21,849	115,369

* As recycling partners were changed in November 2014, there were no data for November and December other than a total of 625 phones donated (i.e. no barcode tracking to allow point of influence to be determined). Therefore the total for 2014 for each point of each influence was estimated by proportion returned for each point of influence in 2013.
