# Supplementary material for: Recycling 115,369 mobile phones for gorilla conservation over a six-year period (2009-2014) at Zoos Victoria: A case study of ‘points of influence’ and mobile phone donations
Source: PLoS One. 2018 Dec 5;13(12):e0206890. doi: 10.1371/journal.pone.0206890 (PMC6281204; doi:10.1371/journal.pone.0206890)
Supplement: S3 Appendix — (PDF) [file pone.0206890.s003.pdf]

S3 Appendix

The Connect – Understand –Act model guided the design and development of the *They're Calling on You* campaign.

1. Select Threatening Process –the conflict mineral coltan used in mobile phones is mined in the Democratic Republic of Congo (DRC) in primate habitats, including national parks, with increased poaching of gorillas and other species.

2. Identify ambassador species - Zoos Victoria cares for western lowland gorillas (*Gorilla gorilla gorilla*), a subspecies not directly impacted by coltan mining in the DRC, however this species serves as an ambassador species for eastern lowland gorilla populations (*Gorilla beringei graueri*) that have been significantly impacted by coltan mining.

3. Identify target audience - Mobile phone users (people from the age of 12 and up) are people within Zoos Victoria's sphere of influence in the zoo and community (e.g. zoo visitors, sponsors). This step prevents development of education programs or campaigns that simply raise awareness about conservation without providing tangible ways for people to contribute positively.

4. Select target behaviour (ACT) – Donation of old mobile phones to the They're Calling on You program at Melbourne Zoo, enabling the Aussie Recycling Program (ARP) to divert unwanted mobile phones from landfill by refurbishing them and selling them, which in turn raised funds for gorilla conservation.

5. Confirm enduring understanding (UNDERSTAND) - Everyday actions such as purchasing or disposing of a mobile phone can impact wildlife both locally and globally.

6. Identify connection opportunity (CONNECT) - Keeper talks provided the best opportunity to conduct story telling whilst weaving in the enduring understanding and delivering the facilitated call to action. These face-to-face encounters can 'hook' visitors to the key message, and zoos have enormous potential to ignite emotional connections between people and wildlife.

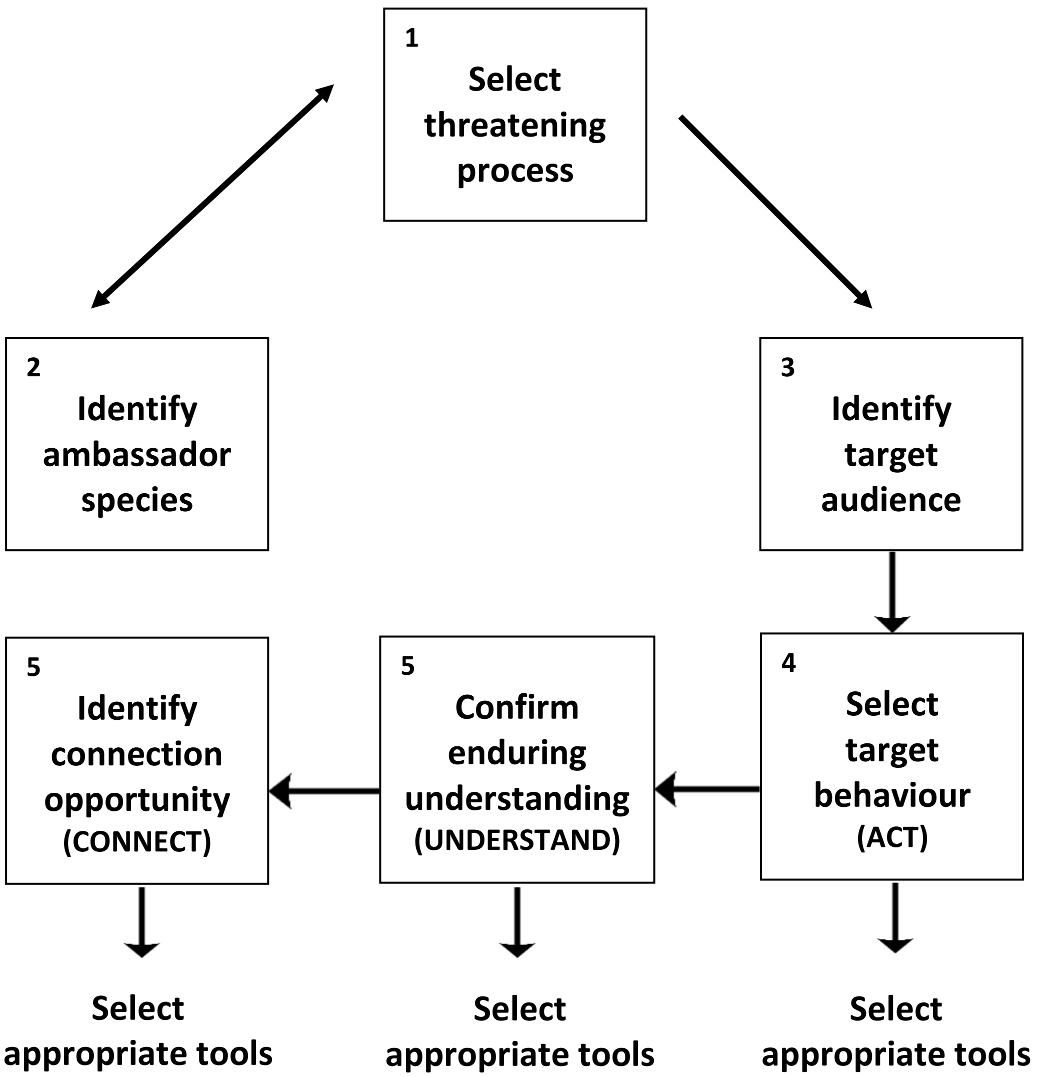

Fig S1. Zoos Victoria 'Connect – Understand – Act' conservation education model.
