# Supplementary material for: Recycling 115,369 mobile phones for gorilla conservation over a six-year period (2009-2014) at Zoos Victoria: A case study of ‘points of influence’ and mobile phone donations
Source: PLoS One. 2018 Dec 5;13(12):e0206890. doi: 10.1371/journal.pone.0206890 (PMC6281204; doi:10.1371/journal.pone.0206890)
Supplement: S4 Appendix — (PDF) [file pone.0206890.s004.pdf]

S4 Appendix

Images showing additional details of three points of influence for the *They're Calling on You* campaign.

A **B**

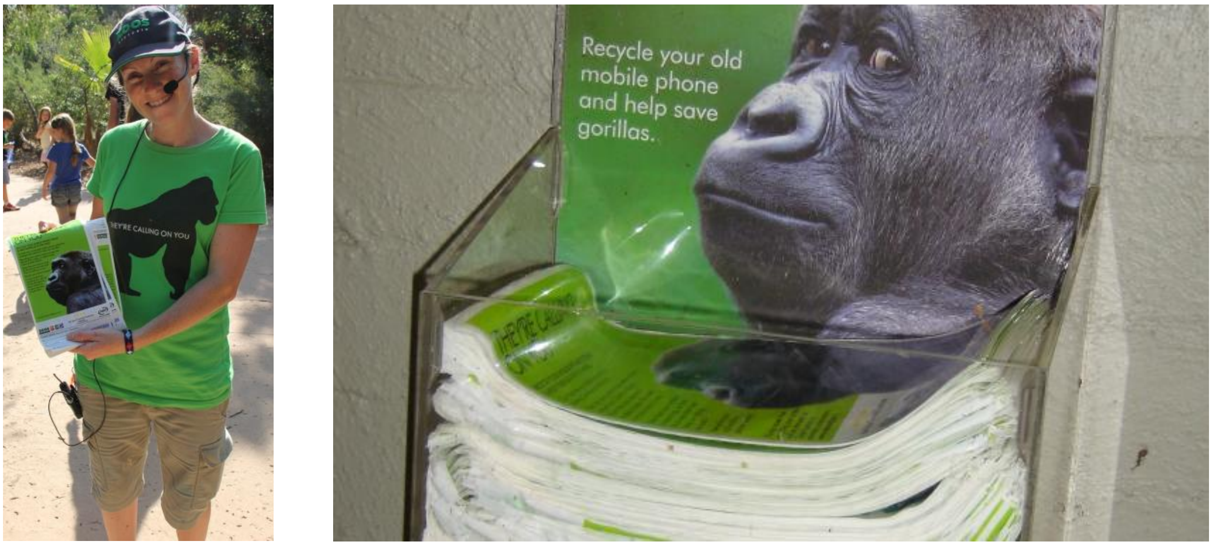

Fig. S1. Two 'inside zoo' points of influence were keeper talks (A) and static displays at the entrance/exit of Melbourne Zoo (B).

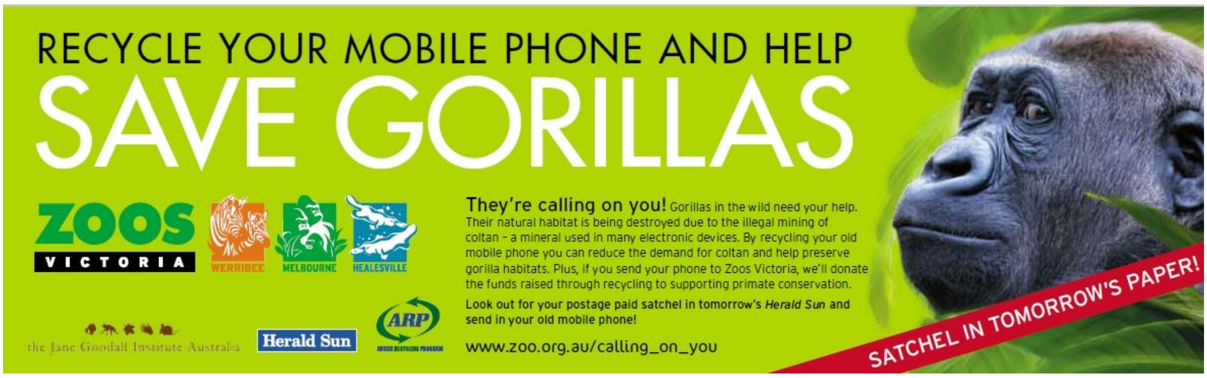

Fig. S2. A *Herald Sun* newspaper promotion advertisement on June 10, 2011, the day before this 'outside zoo' points of influence was launched.
