# Supplementary material for: Recycling 115,369 mobile phones for gorilla conservation over a six-year period (2009-2014) at Zoos Victoria: A case study of ‘points of influence’ and mobile phone donations
Source: PLoS One. 2018 Dec 5;13(12):e0206890. doi: 10.1371/journal.pone.0206890 (PMC6281204; doi:10.1371/journal.pone.0206890)
Supplement: S5 Appendix — (PDF) [file pone.0206890.s005.pdf]

S5 Appendix

Estimated amount of recoverable elements including conflict elements and landfill space saved for the *They're Calling on You* campaign

We calculated the potential recovery weight and value of nine elements (including four conflict elements) contained in the 117,745 mobile phones donated for the six-year period (2009-2014) and included the number of phones donated during the three-month trial period (October-December 2008), based on a groundbreaking study by Christian, Romanov, Romanova, and Turbini in 2014 [22] of metals contained in 85 mobile phones from 2008-2014, as it matched our evaluation time frame (Table 1). Our calculations assume 100% element recovery, which is probably unlikely giving current methods of element extraction. For the four conflict elements (Tin, Tungsten, Tantalum & Gold), their combined weight was 185kg (about 5% of total 3736kg). Iron and copper would be the most common elements present with an estimated combined weight of 3240 kg (about 87% of total).

Estimate of landfill space saved

The physical space required to store the 117,745 mobile phones can be calculated. A current Blackberry is more or less 12 cm x 7 cm x 1 cm = 84 or ~100 cm^3. Multiplying by 117,745 = 11,774,500 cm^3 or approximately or approximately 11.8 m^3. Mobile phones of different sizes never pack that well, so they would be likely to take up somewhere between 12 and 20 cubic meters. This is about the size of a large rubbish truck as shown in Figure S1.

Table 1. Maximum and minimum weights of elements contained in mobile phones, and potential weight and costs of metals contained in the 117,745 mobile phones collected by Zoos Victoria during 2008-2014 (i.e. includes three-month trial period).

**Element**	**Max (g)**	**Min (g)**	**Avg (g)**	**Total (Kg)**[a]
**Iron (Fe)**	50.37	3.37	16.46	1938
**Copper (Cu)**	29.04	5.07	11.06	1302
**Nickel (Ni)**	5.65	0.42	2.03	239
**Zinc (Zn)**	5.95	0.06	0.53	62
**Silver (Ag)**	0.26	0.02	0.09	10
**Conflict Elements**				
**Tantalum (Ta)**	0.23	0.003	0.03	3.5
**Gold (Au)**	0.07	0.006	0.03	3.5
**Tin (Sn)**	1.82	0.43	0.94	110
**Tungsten (W)**	1.21	0.03	0.58	68

[a]This calculation is for the average (g) multiplied by 117,745, then divided by 1000, and then if >10 kg it is rounded down to the nearest kilogram.

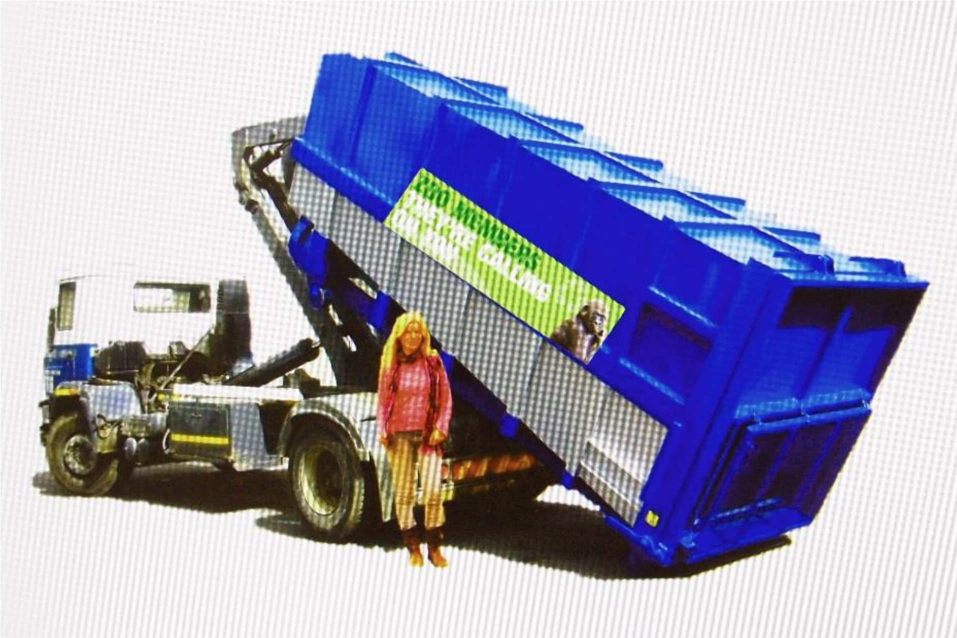

Fig. S1. Image of estimated space required to store mobile phones donated for the period 2009-2014 with a person standing next to the rubbish truck/skip to give an indication of scale.
